# Association study of *CLDN14* variations in patients with kidney stones

**DOI:** 10.1515/biol-2021-0134

**Published:** 2022-02-28

**Authors:** Ihsan Ullah, Khadijah Murtaza, Hafiza Ammara, Misbah, Munir Ahmad Bhinder, Amjad Riaz, Wasim Shehzad, Muhammad Yasir Zahoor

**Affiliations:** Molecular Biology and Biotechnology Section, Institute of Biochemistry & Biotechnology, University of Veterinary & Animal Sciences, Syed Abdul Qadir Jillani (Out Fall) Road, Lahore 54000, Pakistan; Department of Medicine, Services Hospital, Lahore 54000, Pakistan; Department of Human Genetics and Molecular Biology, University of Health Sciences, Lahore 54000, Pakistan; Department of Theriogenology, University of Veterinary & Animal Sciences, Lahore 54000, Pakistan

**Keywords:** cell tight junction, calcium homeostasis, nephrolithiasis, hearing loss, gene association

## Abstract

Claudin-14 protein plays an essential role in regulating calcium ions in the kidney and ear. Two phenotypes, hearing loss and kidney stones, were reportedly associated with variations in the *CLDN14* gene. This study aimed to understand *CLDN14* mutations’ contribution to hearing loss and renal stone formation in a Pakistani cohort. We analyzed *CLDN14* sequence variations in 100 patients, along with healthy individuals, to assess whether specific polymorphisms were associated with the disease. Also, we performed an *in silico* analysis using a mutation database and protein annotation. The rs219779’s genotype CT (*p =* 0.0020) and rs219780’s genotype AG (*p* = 0.0012) were significantly associated with kidney stones. We also found that a novel haplotype, “TA” associated with kidney stone formation, has moderate linkage disequilibrium. The TA haplotype was significantly correlated with a kidney stone risk formation of 3.76-fold (OR (CI 95%) = 3.76 (1.83–7.72)) and *p* = 0.0016 compared to other haplotypes. *In silico* analysis revealed that mutations associated with hearing loss were not correlated with renal stone formation but affected claudin-14 protein stability. We structurally mapped a novel TA haplotype of *CLDN14* that, based on our analysis, likely contributes to the pathogenesis of renal stones.

## 1 Introduction

Tight junctions, also known as zonula occluden, play a significant role in cell-to-cell adhesion in epithelial or endothelial tissues. They act as a physical barrier, continuously sealing the cell–cell junction, allowing controlled transportation of water and solutes across the paracellular space in the renal tubule and epithelium [1]. Tight junctions consist of protein strands interwoven into the junction lipid bilayer, making strong contacts with protein strands of the adjacent cells [1]. Claudins are composed of four transmembrane domains, two extracellular loops, a short cytosolic N-terminus, and longer cytosolic C-terminus. The first extracellular domain is crucial for determining selectivity. Several claudin isoforms in the renal tubule of adults and neonates have been reported to determine the paracellular diffusion pathway’s capacity, selectivity, and permeability [2].

The claudin protein family comprises 27 members, each expressed in specific tissues [3]. The integral membrane protein claudin-14, encoded by the *CLDN14* gene, is a part of tight junctions. The claudin-14 protein binds specifically to the Yes-associated protein (YAP), a novel transcriptional co-activator [4]. Claudin-14 is attached to a neighboring claudin-14 via an extracellular groove, controlling calcium transportation in the junction [5]. Claudin 14 regulates calcium reabsorption in the ascending limb [6,7,8]. Claudin proteins play a vital role in ion regulation; any abnormality in this protein might lead to dysregulation of calcium and other ions [9]. Hou [10] suggested that the tight junction complex at the apex of the reticular lamina requires claudin-14 as a cation-restrictive barrier to maintain the proper ionic composition of the fluid surrounding the basolateral surface of outer hair cells. The calcium-sensing receptor (CASR) and calcium intake may regulate the expression of claudin-14 [11,12,13,14]. Many synonymous variants in the *CLDN14* gene were reportedly linked to kidney stone formation and reduced bone mineral density [15]. Previously, nonsynonymous homozygous mutations in *CLDN14* were correlated with the autosomal-recessive nonsyndromic sensorineural deafness disease [16,17]. The precise role and regulatory mechanism of claudins are not fully explained, and a great deal of work remains [18]. *CLDN14* mutations exhibit two phenotypes that are not reported together. This study aimed to determine the role of the *CLDN14* gene in nephrolithiasis-affected individuals of the local Pakistani population. We performed an *in silico* analysis of *CLDN14* variants by employing different databases for nephrolithiasis and hearing loss phenotypes to determine the role of *CLDN14* variants on gene regulation and protein structural stability.

## 2 Material and methods

### 2.1 Enrolment of patients

One hundred patients from unrelated families were enrolled. Inclusion criteria: each had one or more kidney stones, with calcium being the main stone component. The diagnosis was made using ultrasound, chemistry, and analysis of urine and stone composition (if available). Exclusion criteria: Patients with other kidney and metabolic diseases, nephrosis, diabetes, and heart patients were excluded. Clinicians (physicians) from different districts of Punjab province performed patient enrolment. Patients were recruited from Shaikh Zahid Hospital Lahore, General Hospital Lahore, Services Hospital Lahore, Civil Hospital Bhawalpur, District Civil Hospital Hafizabad, District Civil Hospital Narowal, and some patients were recruited from home via different references. Patient information, such as diet, cast, age, weight, and history of other diseases, was obtained. The control sample was taken from healthy people with no family and personal history of stone or kidney diseases.


**Informed consent:** Informed consent has been obtained from all individuals included in this study.
**Ethical approval:** The research related to human use has been complied with all the relevant national regulations, institutional policies, and in accordance with the tenets of the Helsinki Declaration, and has been approved by the UVAS (Institutional Review Board) IRB Ref: IRB/217/12 and Ethical Review Board of Service Institute of Medical Sciences (SIMS), Lahore Ref No. IRB/2017/334/SIMS.

### 2.2 Sequencing

Genomic DNA was extracted from blood samples using the standard organic methods [19]. Exon 3 and the untranslated regions (UTRs) of the *CLDN14* gene was amplified using a set of primers designed by Primer3 Software [20] ([forward: 5′-CTTGGCTTCATTAGGGCTCC-3′] 60.5°C; [reverse: 5′-GAACCCCTGCCTCCATTGA-3′] 59.5°C). Each amplicon was sequenced in both orientations using Sanger dideoxy chain termination chemistry. Sequencing polymerase chain reaction (PCR) products were separated using ABI PRISM 3700 Genetic Analyser (Foster City, CA, USA). PCR was carried out in the T100^TM^ Thermal Cycler (Bio-Rad, Hercules, CA, USA) by using a 25 µL reaction mixture containing 50 ng DNA, 1× (NH_4_)_2_SO_4_ buffer, 250 µM of each dNTPs, 1.5 mM MgSO_4_, 10 µM of each primer and 2.0 U Taq DNA polymerase. All PCR amplifications were performed at an annealing temperature of 59°C.

### 2.3 Statistical analysis

The analysis of enrolled patients and control data, including allelic and genotypic frequencies expressed as counts (percentages), was performed using the statistical package for social sciences (SPSS) version 20. Using a Chi-square test, Hardy–Weinberg equilibrium (HWE) was performed, which served as a statistical control for systematic genotyping error and population stratification, where *CLDN14* polymorphisms that violated HWE, as indicated by *p*  <  0.05 for in the control group, were not processed for further data analyses. Odds ratios (ORs) with associated 95% confidence intervals (CI) were determined to assess the strength of statistical association, if any, considering allelic, genotypic, recessive, dominant, and log-additive models by the same SNPstats program [21]. The pairwise linkage disequilibrium and haplotype analysis for *CLDN14* polymorphisms were conducted using the Haploview program [22]. The Bonferroni correction for multiple testing was performed to calculate ORs and associated *p*-values for genotype and haplotype associations between *CLDN14* polymorphisms and kidney stones.

### 2.4 *In silico* analysis

We performed an analysis of variants associated with renal stone formation and hearing loss using different resources: Human Genome Mutation Database (HGMD) [23], ClinVar, Exome Variant Server, and literature [24]. The amino acid conservation, mutation, and domain specificity analyses were carried out using Clustal Omega alignment of claudin-14 and orthologous sequences from human, mouse, chicken, and frog downloaded from the Ensemble database. The crystal structure of mouse claudin-14 large domain, claudin-19 (3X29:A), was obtained from the Protein Databank (https://www.rcsb.org/). PyMol [25] was used to analyze the claudin-19 structure by studying mutational interactions and structural stability.

## 3 Results

### 3.1 Cohort characteristics

The average age, body weight, and gender percentage of the groups are presented in Table 1. In the patient group, the average male age was 20.7 years compared to that of the female was 22.5 years. The average male weight was 62 kg compared to that of females at 57.2 kg. Overall, the patient’s weight and age were lower than those of controls, suggesting that the stone phenotype appears at a younger age (young-onset).

**Table 1:** Clinical and genetic characteristics of patients

	Characteristic	No.	Average age (Year)	Average weight (kg)	Percentage
Normal (100)	Male	63	29.42	66.1	63
	Female	37	33.34	52.35	37
Patients (100)	Male	72	20.7	62	72
	Female	28	22.5	57.2	28

### 3.2 Sanger screening

**Figure 1:**
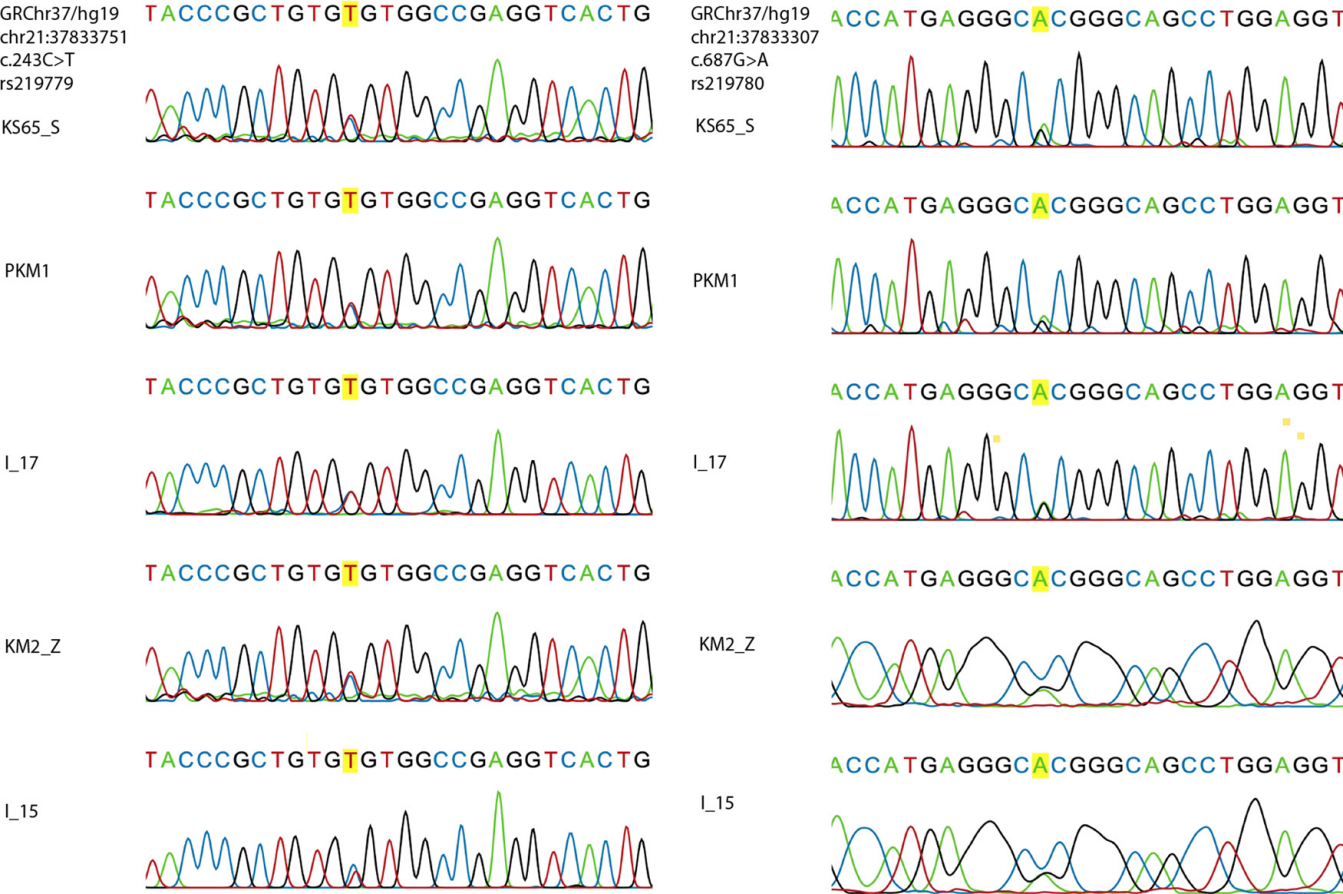
Sanger sequencing of *CLDN14* in patients. Rows are annotated with coordinates of SNPs rs219779 and rs219780, respectively, in five samples: KS_11, KS_13, KS_9, KS_2, and KS_15.

We found two SNPs, rs219779 (C > T) and rs219780 (G > A), through Sanger sequencing of *CLDN14* coding exon 3 and UTRs (Figure 1 and Table 2) in patients with kidney stones. The patients are heterozygous for specific alleles, i.e., the genetic score less than 1 [23] indicated the changes are polymorphisms and not monogenic and less pathogenic. The allelic and genetic frequencies for all the *CLDN14* polymorphisms did not violate the HWE in the control group.

**Table 2:** Association of *CLDN14* rs219779 and rs219780 variants with nephrolithiasis disorder considering allelic and genotype frequencies

Group	Allele	Case	Control	OR (CI 95%)	*p* value*
rs219779	C	135 (0.68)	159 (0.80)	Referent	0.0368
	T	65 (0.32)	41 (0.20)	1.8672 [1.1867–2.9378]	
rs219780	G	135 (0.68)	160 (0.80)	Referent	0.0256
	A	65 (0.32)	40 (0.20)	1.9259 [1.2211–3.0375]	
rs219779	CC	35 (0.35)	60 (0.60)	Referent	
	CT	65 (0.65)	39 (0.39)	**2.8571 [1.6067–5.0809]**	**0.0020**
	TT	0 (0)	1 (0.01)	0	2.54
rs219780	GG	35 (0.35)	61 (0.61)	Referent	
	AG	65 (0.65)	38 (0.38)	**2.9812 [1.6742–5.3086]**	**0.0012**
	AA	0	1 (0.01)	0 (0)	2.556

OR: odds ratio; CI: 95% confidence interval; *p values reflect adjustment for age and gender. Bonferroni correction for multiple testing was applied (*p* value threshold= 0.01). Statistically significant *p* values (<0.01) and associated OR values are highlighted in bold.

The genotypes rs219779 CT (OR:2.8571, *p*: 0.0020) and genotypes rs219780 AG (OR:2.9812, *p*:0.0012) have significant association with kidney stones (Table 2). We have only genotype TT (rs219779) and AA (rs219780) in controls but not in patients. So, we cannot evaluate recessive and dominant genetic models.

### 3.3 Haplotype analysis and linkage disequilibrium

The haplotype analysis was conducted on genetic data of the patient and control groups using SNPstats software [21]. The promising candidate haplotype was TA as evidenced by the *p*-value of 0016, Odds Ratio of 3.76, and 95% CI [1.83–7.72] (Table 3). The other haplotypes exhibited higher allele frequencies in the control samples with a low *p*-value (Table 3). Furthermore, the TA haplotype frequency was 0.1146 in the control group and 0.2291 in patients, suggesting that the association between this haplotype and kidney stone disorders’ etiology was statistically significant. The global haplotype association *p*-value was 0.0019. Pairwise LD analysis and haplotype plot construction demonstrated moderately significant D’ measures between two pairs of both SNPs, suggesting that these two loci pairs may be linked together. Furthermore, the trend of association persisted for the haplotype to check the combined effect of LD analysis and shows a moderate effect of both SNPs because both fall in the same block with a score of 53 as shown in Appendix Figure A2.

**Table 3:** Association of kidney stone risk with *CLDN14* genetic variants considering haplotype analysis

Haplotypes	Case (freq.)	Control (freq.)	Odds ratio [95%CI]	*p*-value*
CG	0.5791	0.7096	1.00	—
TA	0.2291	0.1146	**3.76 (1.83–7.72)**	**0.0016**
TG	0.0959	0.0904	2.13 (0.91–4.96)	0.328
CA	0.0959	0.0854	2.53 (1.04–6.16)	0.172

OR: odds ratio; 95% CI: 95% confidence interval; *p values reflect adjustment for age and gender. Bonferroni correction for multiple testing was applied (*p* value threshold= 0.01). Statistically significant *p* values (<0.01) and associated OR values are highlighted in bold.

### 3.4 Conservational annotation of reported mutations

**Figure 2:**
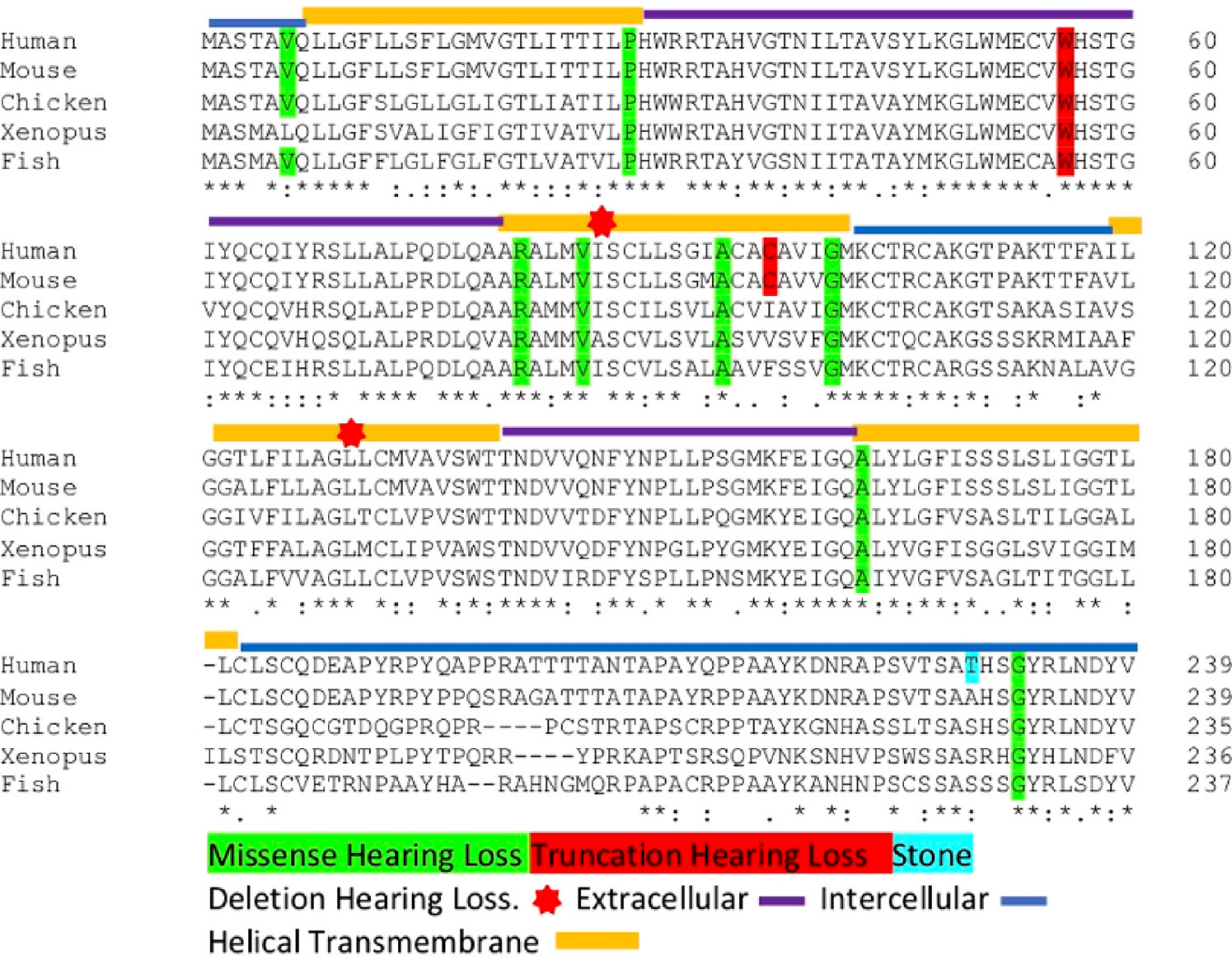
Clustal alignment of human, mouse, chicken, frog, and fish. Mutations are coloured according to their type, whereas domains are separated by lines.

**Figure A1:**
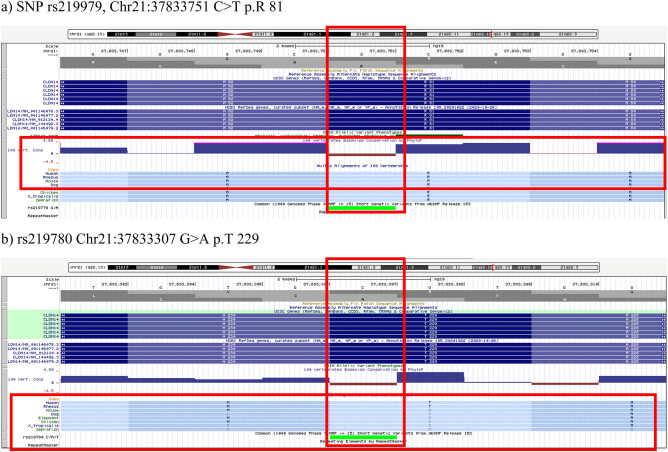
Genomic position of both SNPs and conservation of amino acid: (a) SNP rs219979, Chr21:37833751 C >T p.R 81; (b) rs219780 Chr21:37833307 G >A p.T 229.

Numerous mutations in *CLDN14* were reportedly associated with hearing loss, and all exhibited higher genetic scores, according to the American College of Medical Genetics and Genomics guidelines. Herein, we conducted a conservation analysis of *CLDN14* sequences with both hearing loss- and kidney stone-associated mutations using the HGMD database and the literature (Figure 2 and Figure A1). The *CLDN14* orthologs were present up to fish and not in lower organisms. Importantly, we found almost all hearing loss-associated mutations located in the helical transmembrane domain, critical for protein stability and ion transport. In contrast, kidney stone-associated mutations were located in the regulatory regions and UTRs.

### 3.5 Protein structural analysis

**Figure 3:**
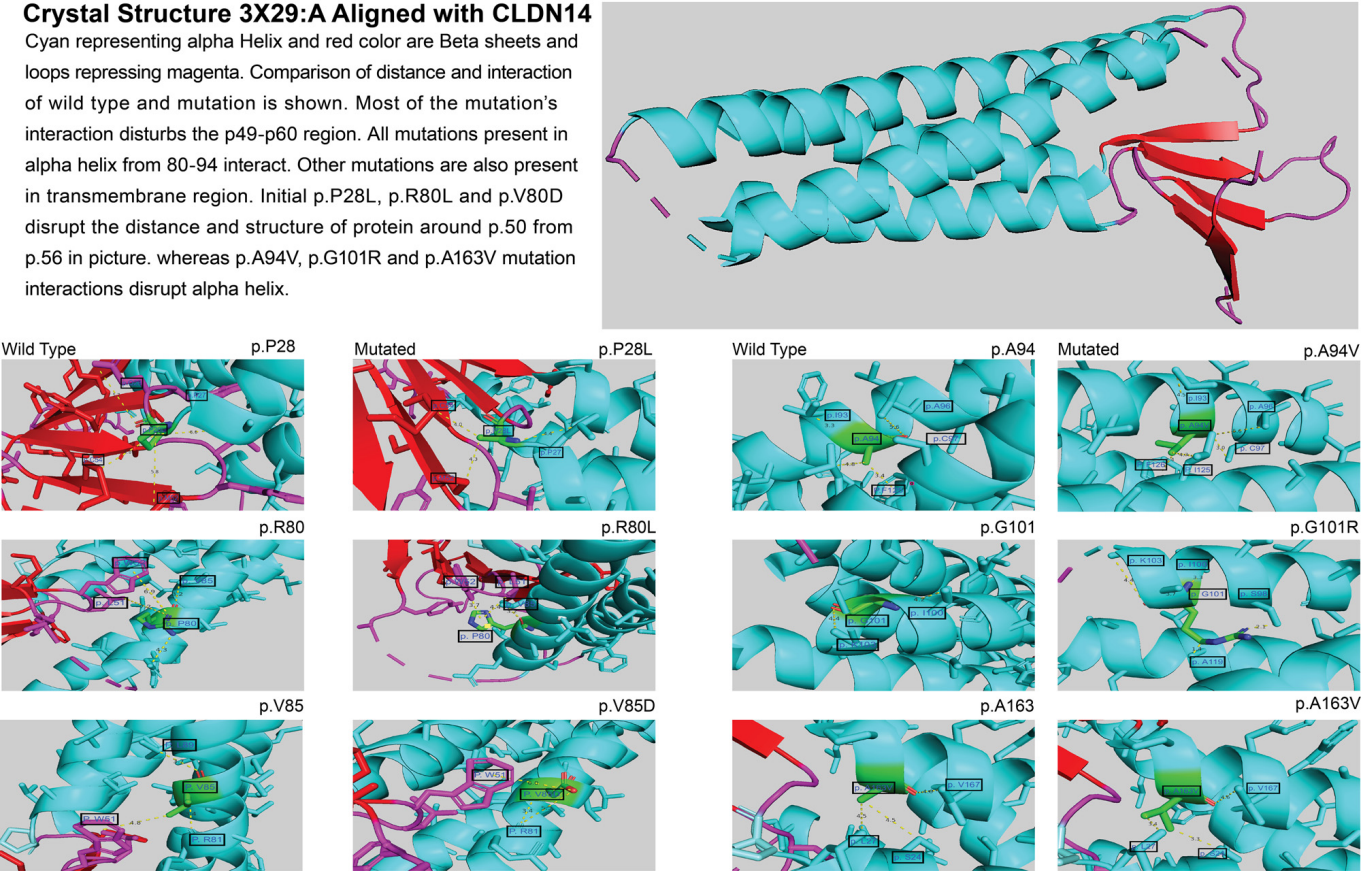
Crystal structure mutation annotations using PyMol. Mutations (shown in dots) were mapped onto the structure of claudin-19 with distances in Å. Amino acid interactions are compared between wild type and mutants. The mutated amino acid is highlighted in green and is connected via dotted lines to the interacting amino acid.

The mouse claudin-19 (3X29:A) was used for protein structure analysis as it is closely related to claudin-14. Based on our amino acid alignment (Figure 2), the mutation is conserved in the structure. We found structural abnormalities in the mutated protein by comparing protein simulations and distance measurements of normal and mutated proteins (Figure 3). Strong interactions are indicated by bond lengths between 1.5 and 4.0 Å [26]. Using PyMol, we generated mutations in the following amino acids’ crystal structure: p.P20, p.R80, p.V85, p.A94, p.G101, and p.A163, all of which were present in the transmembrane region. In these mutants, amino acid interactions with neighboring amino acids changed. The most notable changes were found in the transmembrane region (amino acids 82-102), which interact with amino acids 50-56 (Figure 3). Kidney stone-associated mutations were not found in or around regions where hearing loss mutations were present.

## 4 Discussion

**Figure A2:**
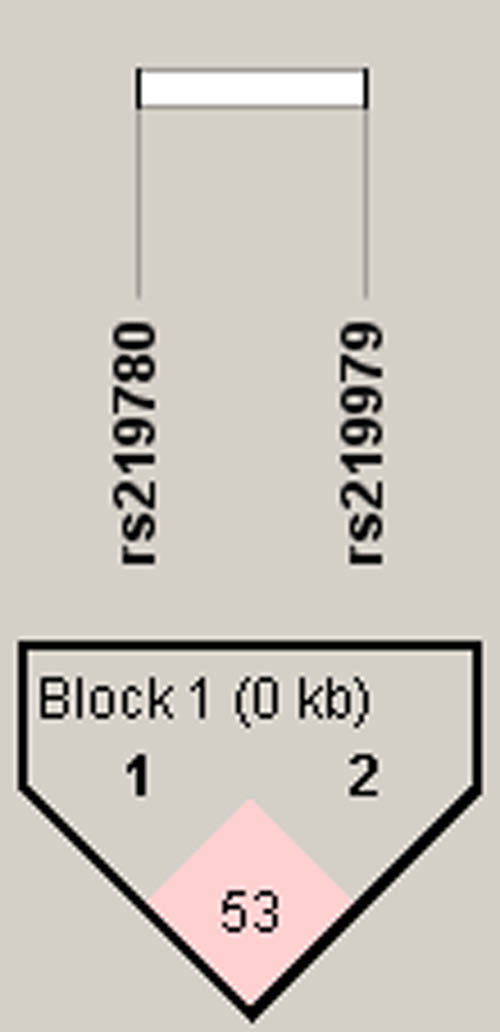
Linkage disequilibrium (LD) analysis of rs219979 and rs219780 SNPs of the *CLDN14* gene.

Claudin-14 is an integral membrane protein with a role in calcium regulation and ion homeostasis. Several *CLDN14* variants have been associated with hearing loss and renal stone formation. Wilcox et al. [16] reported that recessive inheritance of *CLDN14* variants was associated with hearing loss in a Pakistani cohort. Thorleifsson et al. [15] illustrated that the rs219780 SNP was linked to renal stone formation in patients from a large cohort study (*p*-value = 4.0 × 10 (−12) of Iceland and the Netherlands. Thorleifsson’s study also shows the association of rs219779 with kidney stones. Interestingly, rs219779 was reportedly involved in higher serum parathyroid hormone (PTH), which regulates calcium homeostasis [27]. Although these two SNPs were reported associated with kidney stone risk in the Indian population [28], the specific haplotype was not identified. In the Guha study, rs219780 allele and genotype frequency were *p* < 0.001 compared to our allele A (G referent) *p* = 0.0256 and genotype AG *p* = 0.0012. Their LD analysis of three SNPs (rs219777, rs219778, and rs219780) do not suggest any strong findings, but our findings suggest moderate association in one block (Figure A2). Furthermore, our study suggests that rs219779 have a stronger association with kidney stone compared to previous studies suggesting rs219780 [15,28]. We identified a novel TA haplotype of *CLDN14* SNPs, rs219779 and rs219780, and its association with the renal stone formation, which is not previously studied in *CLDN14* studies. The significant *p*-value (0.0016) of the TA haplotype in our study suggests a strong association with kidney stone risk formation. The present study’s findings confirmed those in previous work [6], showing that rs219779 and rs219780 were in linkage disequilibrium; this explains the predictive role of the TA haplotype.

This suggests that mutations in intergenic parts like UTRs and enhancer regions indirectly involved in cell signaling can lead to increased calcium levels in specific tissues and contribute to stone formation. Some studies have also described the role of somatic variation of *CLDN14* in cancer [29].

We assumed stone-associated variations could make *CLDN14* susceptible to microRNA silencing in the regions that control gene regulation. Calcium levels control the silencing function of two microRNAs (miR-9 and miR-374). miR-9 and miR-374 repressed the translation of claudin 14 protein by silencing the transcript in kidneys [30]. Nishi et al. [31] reported that this protein had a similar function in many species but the difference existed in ion regulation due to the molecular location of regulatory domains. These data indicated that protein function is evolutionarily adapted and can change depending on epigenetics and environmental factors like diet. These changes can affect the signaling cascade and gene regulation as observed in cancer [32].

Overall, we identified the TA haplotype and its association with kidney stone formation but no additional monogenic or genetically strong mutations in our cohort. We supplemented our study with an *in silico* analysis. We determined that amino acids 49-60 were crucial for extracellular and transmembrane interactions. Our structural analysis suggests that the transmembrane region is vital for structural stability and function (Figure 3) [32]. Also, our *in silico* studies showed that the mutations associated with hearing loss were highly conserved.

## 5 Conclusion

We found a novel TA haplotype (*p* = 0.0016) of rs219779 and rs219780 SNPs, both of which were previously reported to correlate with kidney stone formation in different populations in separate studies. Furthermore, our study suggested that hearing loss was associated with genetically strong monogenic mutations in *CLDN14* causing protein instability, whereas stone-associated variations of *CLDN14* affected gene regulation.
